# An Improved Wavelet Packet-Chaos Model for Life Prediction of Space Relays Based on Volterra Series

**DOI:** 10.1371/journal.pone.0158435

**Published:** 2016-06-29

**Authors:** Lingling Li, Ye Han, Wenyuan Chen, Congmin Lv, Dongwang Sun

**Affiliations:** 1 Department of Electrical Engineering, Hebei University of Technology, Tianjin, China; 2 Department of Electrical Engineering, National Chin-Yi University of Technology, Taichung, Taiwan; 3 Jiangxi Electric Power Design Institute, Jiangxi, China; Beijing University of Technology, CHINA

## Abstract

In this paper, an improved algorithm of wavelet packet-chaos model for life prediction of space relays based on volterra series is proposed. In the proposed method, the high and low frequency time sequence components of performance parameters are obtained by employing the improved wavelet packet transform to decompose the performance parameters of the relay into multiple scales. Then the optimization algorithm of parameters in volterra series is improved, and is used to construct a chaotic forecasting model for the high and low frequency time sequence components gained by the wavelet packet transform. At last, the chaotic forecasting results of the high and low frequency components are combined by taking the wavelet packet reconstruction approach, so as to predict the lifetime of the studied space relay. The algorithm can predict the life curve of the relay accurately and reflect the characteristics of the relay performance with sufficient accuracy. The proposed method is validated via a case study of a space relay.

## Introduction

Aerospace relay plays an important role in the fields of national defense and space field, and its reliability is related to the safety of the aerospace field [1, 2, 3]. Recently, wavelet packet as a new time-frequency analysis method based on frequency band has been extensively applied to signal processing, such as time-frequency analysis, weak signal extraction and non-stationary time series analysis [4, 5]. The performance parameters of space relay are weak signals and non-stationary time series signals. Therefore, it is natural to use the wavelet packet transform [6, 7] to process the performance parameters of aerospace relay. There are two kinds of algorithms to select the best basis in the wavelet packet transform: (1) Coifman and Wickerhauser [8] proposed the simple and effective BBS algorithm based on entropy in 1992. This algorithm utilized the complete wavelet package binary tree structure to select the best basis by bottom-up, which was also called “static pruning”. (2) Volkan kumbasar proposed the BFA method [9] which listed all possible binary tree structures, making comparison among these structures which meet the complete reconstruction conditions. The approach to obtain the best basis was to select a minimum group of structure in the metric functions. The traditional relay life models [10, 11] are based on the linear autoregressive model [12, 13] and linear autoregressive moving model [14, 15], such as vector auto regressive model, bilinear model and threshold auto regressive model. These models have a better prediction capability for linear systems, while for the non-stationary performance parameters of the space relay, the above linear models could cause significant errors in relay life prediction. Chaos theory [16, 17] as the new research method of nonlinear science is put forward. Due to the environment loads, the performance parameters of relay have some random and chaotic properties. Therefore, the prediction result is also unsatisfactory if just the chaos model [18, 19] is employed for the lifetime prediction of the relay.

In this paper, an improved wavelet packet transform and an improved volterra series [20, 21] of chaos models are proposed, and both are combined to predict the lifetime of relay. Firstly, the performance parameters generated during the work of relay are multi-scale decomposed by using the wavelet packet transform, getting the high and low frequency timing components of performance parameters. Then, the improved chaotic prediction method based on volterra series to model and forecast the time components and obtain the chaos prediction results of each high and low frequency component. Finally, the chaotic prediction results are combined by use of the wavelet packet reconstruction to obtain predicted life of relay.

## Wavelet packet transform

Real coefficient wavelet packet transform is frequently employed. Consequently, it is enough to consider only real coefficient filter here. The relationship between the scaling and wavelet functions is defined as follows:

$$\begin{array}{l} \phi \left(t\right)=\sqrt{2}\sum_{k\in z}h_{0k}\phi \left(2t-k\right)\\ \psi \left(t\right)=\sqrt{2}\sum_{k\in z}h_{1k}\phi \left(2t-k\right) \end{array}\;,$$ (1)

In the above formula, *h*_0*k*_ and *h*_1*k*_ denote the coefficients of high-end filter in the multi-resolution analysis respectively.

The two-scale Eq (1) demonstrates that the wavelet basis *ψ*(*t*) can be obtained by the linear combination of translation and scaling of *ϕ*(*t*).

Given a fixed scale, a series of recursive functions are defined as follows:

$$W_{2n}\left(t\right)=\sqrt{2}\sum_{k\in z}h_{0k}W_{n}\left(2t-k\right)$$ (2)

$$W_{2n}{}_{+1}\left(t\right)=\sqrt{2}\sum_{k\in z}h_{1k}W_{n}\left(2t-k\right),n=0,1,2,\ldots ,N\;,$$ (3)

where the sequence of function *W*_*n*_(*t*)(*n* = 0,1,2,…, *N*) are the wavelet packet determined by *W*_0_ = *ϕ*(*t*).

**Fig 1:**
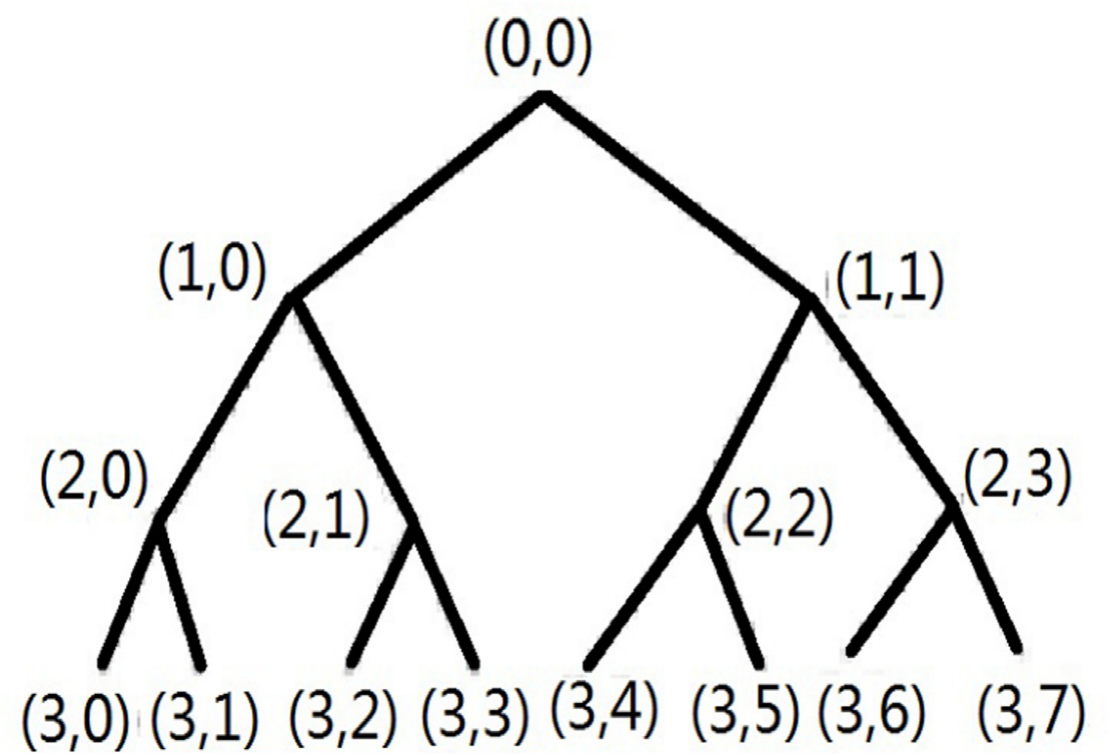
The schematic diagram of the three-level wavelet packet decomposition tree.

The schematic diagram of three-level wavelet packet decomposition tree is shown in Fig 1, the left sub-node is the sub graph of the low frequency approximation of the parent node. The right sub-node is the sub graph of the high frequency details of the parent node. The node (0, 0) denotes the original signal to be decomposed.

## Selection of the best wavelet packet basis

### 3.1. Cost Functions

Since *w*_*j*_ has different analytic methods, i.e., different orthogonal bases, the “best basis” needs to be chosen under the different scales of the wavelet packets transform. For a given vector, the minimum cost means the most effective expression, the basis which meets the minimum cost is the “best basis”.

For the best basis selection problem [22, 23], the corresponding cost function should be chosen according to the different research contents. At present, the frequently employed cost function bases on the Shannon information entropy [24], which is not practical for the real-time requirements of the space relay. In order to satisfy the real time and accuracy property of space relay, a novel cost function based on the improved Shannon information entropy is proposed:

$$M\left(x\right)=\left(x_{i}-u\right)/S\;,$$ (4)

where *u* = (1/*n*)∑*x*_*i*_, $S=\left(1/n\right)\sum_{j}\left(\left|x_{j}\right|/\left\|x\right\|\right)$.

The improved cost function based on the minimum variance idea determines the optimal decomposition tree of the wavelet packet. It is dispensable to calculate the large amount of computation, the calculation possesses characters of simple and high real time.

### 3.2. The improved wavelet packet best basis selection algorithm

The wavelet packet best basis selection algorithm can be classified into two categories:

In BBS algorithm, the number of the bottom nodes grows by 2^∧^*n* when the decomposition level turn into *n* + 1, which leads to the number of selected terminal node cannot be controlled. The BFA algorithm cannot be applied to large scale operations because its high computational complexity. Based on the above, the number of components of the high and low frequency entirely depends on the decomposition level and must be 2^∧^*n* in the whole decomposition structure of wavelet packet.

In this paper, a novel algorithm is proposed for the selection of the best basis based on the cost function of the improved Shannon information entropy [25]. The proposed algorithm sets the number of end node *R* as a search constraint condition, and does not need to introduce a lot of extra calculation. Based on the choice of the root node and the child node, the algorithm takes the competition method to solve the optimal output problem. The algorithmic steps are:

Step1: Initialization of the data, the root node $w_{0}^{0}$ is decomposed into two decomposable child node $w_{1}^{1}$ and $w_{2}^{1}$. Set initial value: the decomposable point *n*_1_ = 2, and the indecomposable point *n*_0_ = 0.

Step2: If $w_{j}^{i}$ is a decomposable node, and 2*n*_1_ + *n*_0_ < *R*.

1. For *i* = 1; *i* = *i* + 1, until all nodes are marked as terminal nodes;
2. For *j* = 1:2^*i*^, the node $w_{j}^{i}$ is decomposed into two child nodes $w_{p}^{i+1}$ and $w_{p+1}^{i+1}$;
3. The metric function values of $w_{j}^{i}$ and $w_{p}^{i+1}+w_{p+1}^{i+1}$ are calculated according to the Eq (3), denoted as $f_{\text{cos}\;t}^{i}$ and $f_{\text{cos}\;t}^{i+1}$;
4. If $f_{\text{cos}\;t}^{i}\geq f_{\text{cos}\;t}^{i+1}$, delete $w_{j}^{i}$, then mark $w_{p}^{i+1}$ and $w_{p+1}^{i+1}$ as the decomposition point, else delete $w_{p}^{i+1}$ and $w_{p+1}^{i+1}$, mark $w_{j}^{i}$ as the end node.

Step3: Repeat the previous step for the separable nodes of next level, until 2*n*_1_ + *n*_0_ > *R*, then enter the same level competition.

In the above steps, $w_{0}^{0}$, $w_{1}^{1}$ and $w_{2}^{1}$ are nodes in the wavelet packet decomposition tree.

The difference value of the cost function $g_{add}^{k}$ between each node and its child node determines which nodes are decomposed when faced with competition at the same level. The larger $g_{add}^{k}$ is, the smaller value of the cost function of the child nodes is. Under the condition that best basis meet the constraint conditions, in order to obtain the minimum value of cost function in the final search, the first *n* − *m* larger nodes of $g_{add}^{k}$ should be decomposed.

## The determination of the phase space reconstruction parameters in the Chaos model

The traditional method of calculating the phase space reconstruction parameters [26, 27] is to optimize the delay time *τ* and the embedding dimension *m* separately. Although this method can effectively approximate the actual delay time and the actual embedding dimension, it does not take into account the relevance between the delay time and the embedding dimension. Consequently, the calculated delay time and embedding dimension have notable error. At present, the C-C method [28] is a joint optimization method, which optimizes *τ* and *m* together rather than separately and takes into account the interaction between *τ* and *m*. Hence the prediction validity of C-C method will be higher than that of the single optimization method.

However, the C-C method has some shortcomings as follows:

1. In fact, the first zero point of ${\bar{S}}_{2}\left(t\right)$ is not equivalent to the first local minimum point of $\Delta {\bar{S}}_{2}\left(t\right)$, and for the time series of time period is *T*, *t* = *KT* is the zero point of ${\bar{S}}_{2}\left(t\right)$, and it is likely to be the first zero point of ${\bar{S}}_{2}\left(t\right)$, at the same time, it is the global minimum point of *S*_2*cor*_(*t*). Consequently, the obtained conclusions are contradictory.
2. Ideally, the global minimum of *S*_2*cor*_(*t*) is the optimal embedding window *t*_*w*_. In fact, there are several local minimum points which are very close to the global minimum points. These local minimum points can interfere with the interpretation of the global minimum point, and will directly affect the performance and quality of phase space reconstruction.

Given these facts, a joint optimization method of C-C and modified Cao is proposed to determinate the parameters of phase space reconstruction. The main steps of the implementation methods are:

1. Use the C-C method to obtain the curve of *S*_2*cor*_(*t*), theoretically the global minimum point of the *S*_2*cor*_(*t*) is the optimal embedding window. However, there are several local minimum points are exceedingly close to the global minimum points, the interpretation of the global minimum point is interfered.
2. Search all the local minimum points and the global minimum points in the curve of *S*_2*cor*_(*t*). Then define the neighborhood *W*_*i*_(*T*_*i*_) of the best delay time *t* centering on *T*_*i*_, where *i* is the number of the global minimum points and local minimum points.
3. In the neighborhood of *W*_*i*_(*T*_*i*_), all of the value of *t* are likely to be the best delay time. Sort the quasi time delay value of the neighborhood *W*_*i*_(*T*_*i*_) sequentially to obtain the quasi time delay sequence {*T*_*j*_}.
4. Take *T*_*j*_ in the quasi time delay sequence {*T*_*j*_} in turn and plug the different embedding dimension *d*_*i*_ into the *E*(*d*) of Cao method, then obtain the sequence *E*(*T*_*j*_, *d*_*i*_) corresponding to (*T*_*j*_, *d*_*i*_).
5. Horizontal comparison, calculate the values of *E*_1_(*d*_*i*_) for all *d*_*i*_ based on different values of *T*_*j*_. After calculation, find out the value of *E*_1_(*d*_*i*_) that is closest to one under the different value of *T*_*j*_.
6. Vertical comparison, find out the value of *E*_1_(*d*_*i*_) that is closest to one for all of the time delay *T*_*j*_. As a result, the time delay and the embedding dimension corresponding to the *E*_1_(*d*_*i*_) are the optimal phase space reconstruction parameters. Since this algorithm employ the joint optimization method, the determined space reconstruction parameters can characterize the actual situation more accurately.

## Chaos forecasting model based on Volterra series and its improved algorithm

### 5.1. Volterra series

Theoretical research and practical experience indicate that: volterra series can be employed to characterize the large number of nonlinear systems in most cases. Therefore, volterra series can be adopted to construct the prediction model of chaotic sequence. Assume that the input of the nonlinear discrete system [29] is defined as below: *Y*(*n*) = {*z*(*n*), *z*(*n*−1),…*z*(*n*−*N*−1)}, correspondingly, output is defined as below: $y\left(n\right)=\hat{z}\left(n+1\right)$. Based on the definition, the volterra series expansion formula of the nonlinear system function:

$$\begin{array}{l} \hat{z}\left(n+1\right)=F\left(Y\left(n\right)\right)\\ =r_{0}+\sum_{m=0}^{+\infty }r_{1}\left(m\right)z\left(n-m\right)+\\ \sum_{m_{1}=0}^{+\infty }\sum_{m_{2}=0}^{+\infty }r_{2}\left(m_{1},m_{2}\right)z\left(n-m_{1}\right)z\left(n-m_{2}\right)\\ +\sum_{m_{1}=0}^{+\infty }\sum_{m_{2}=0}^{+\infty }\ldots \sum_{m_{p}=0}^{+\infty }r_{p}\left(m_{1},m_{2},\ldots ,m_{p}\right)z\left(n-m_{1}\right)z\left(n-m_{2}\right)\ldots z\left(n-m_{p}\right)+\ldots \end{array}$$ (5)

where *F*(*Y*(*n*)) is the system evolution model, and *r*_*p*_(*m*_1_,*m*_2_,…,*m*_*p*_) is a p-order volterra core. This kind of infinite series expansion can hardly achieve in practical applications. Therefore, it is necessary to use the finite sum form of finite truncated. At present, the most frequently employed two—degree—truncated sum form is:

$$\hat{z}\left(n+1\right)=r_{0}+\sum_{m=0}^{N_{1}-1}r_{1}\left(m\right)z\left(n-m\right)+\sum_{m_{1}=0}^{N_{2}-1}\sum_{m_{2}=0}^{N_{2}}r_{2}\left(m_{1},m_{2}\right)z\left(n-m_{1}\right)z\left(n-m_{2}\right)$$ (6)

Since the limited nonlinear approximation capability of the two-order volterra series, it has a very low accuracy of prediction. Therefore, the three-order volterra series is adopted to deal with the relay performance parameters.

### 5.2. The improved optimization algorithm for parameters of Volterra series

An adaptive filter with excellent performance cannot be separated from the effective adaptive algorithm, which can guarantee the fast convergence characteristics of the approximate structure more effectively. At present, the frequently employed adaptive algorithm is variable step-size least mean square type algorithm (SVS-LMS) [30, 31] based on sigmoid function:

$$e\left(n\right)=d\left(n\right)-x\left(n\right)\omega^{T}\left(n\right)$$ (7)

μ(n)=β(1/(1+exp(−α|e(n)|))−0.5) (8)

ω(n+1)=ω(n)+μ(n)e(n)x(n), (9)

where *d*(*n*) is the desirable output of a M-order adaptive filter, *x*(*n*) is the input vector at the *n* moment, *ω*(*n*) is the weight coefficient vector of the filter at the *n* moment, *μ*(*n*) is an adaptive adjustment step size.

In the SVS-LMS algorithm, the sigmoid function is too complex and does not have the characteristic of slow change when the error is close to zero, which make the SVS-LMS algorithm still has a large step change in the steady state.

In this paper, an improved SVS-LMS algorithm based on the cost function of Shannon information entropy is proposed. Assume that the entropy of the input vector at the *n* moment is:

$$M\left(x\right)=-\sum_{i}P_{i}\;\text{log}\;P_{i}\;,$$ (10)

where *P*_*i*_ = |*x*_*i*_|^2^/‖*x*‖^2^, and when *P* = 0, *P*log*P* = 0.

Assume that the number of input vectors is *q* at the *n* moment, take *q* output vectors *d*(*q*) immediately before *n* moment and calculate the entropy *M*(*d*) of the output vector according to the definition of entropy.

Define the difference between the entropy of the output and input sequences:

e(n)=M(d)−M(x), (11)

then improve the SVS-LMS algorithm by employing Eq (11) to substitute the Eq (7). The concept of entropy is integrated into the SVS-LMS algorithm, which makes up for the shortage of the traditional SVS-LMS algorithm and also improves the computational complexity and accuracy of the algorithm. All the algorithms are implemented by the Matlab7.1 software.

## Experimental results

Based on the analysis of the space relay failure mechanism, the main performance parameters which can effectively characterize the space relay failure are: super-path time, bounce duration, release time, contact resistance and the dynamic process of the peak voltage. Choosing super-path time as the processing data sequence of the forecast model in this paper.

**Fig 2:**
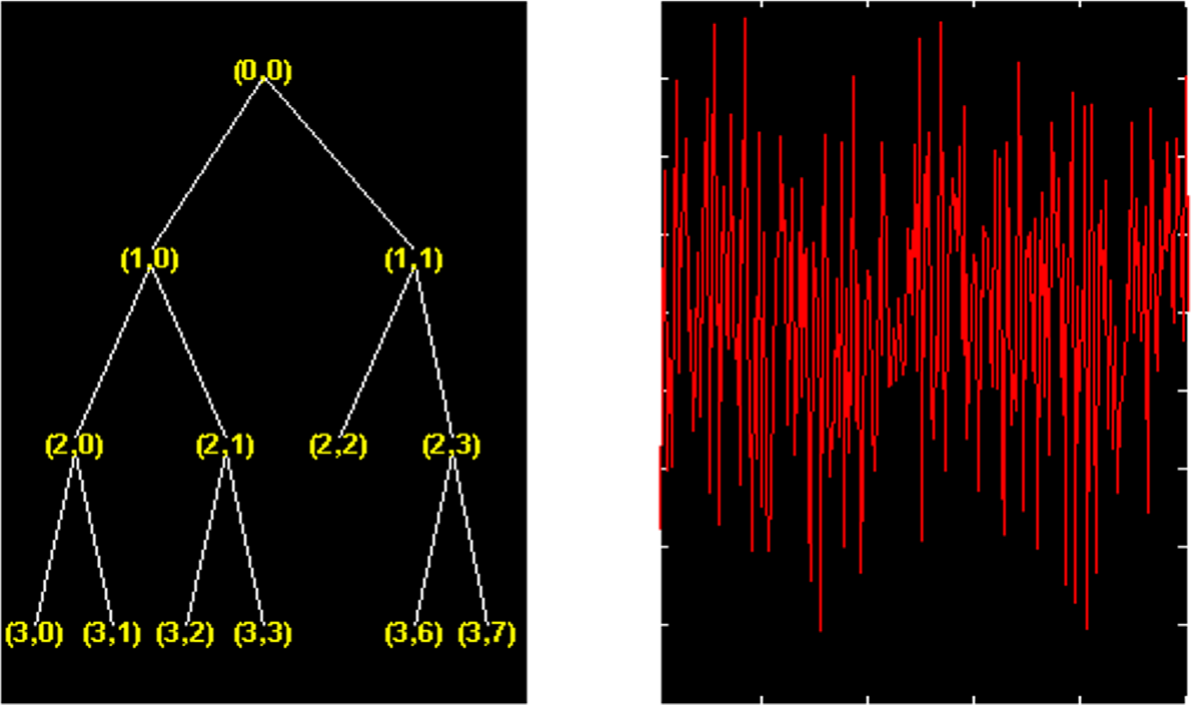
Optimal wavelet packet decomposition tree.

Select 1001 super-path time series points, then employ the wavelet packet to decompose the super-path time series and the decomposition tree of the optimal wavelet packet basis is shown in Fig 2.

**Fig 3:**
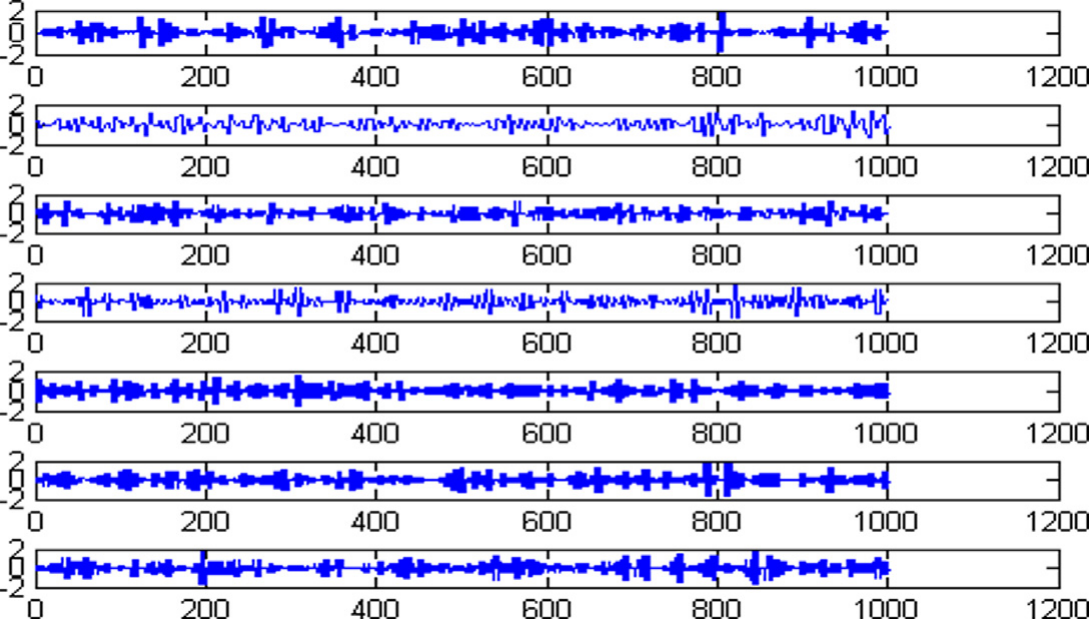
The high and low frequency components of wavelet packet decomposition.

Decompose the super-path time series points with the wavelet packet decomposition on a scale of 3 to get the high and low frequency components, which is shown in Fig 3.

Calculate the obtained eight high and low frequency components’ Lyapunov index values respectively, as shown in Table 1.

**Table 1:** The Lyapunov index values of eight high-low frequency component.

**Component**	(3,0)	(3,1)	(3,2)	(3,3)	(3,4)	(3,5)	(3,6)	(3,7)
** Index values**	0.0031	0.1052	0.2145	0.1987	0.3014	0.2907	0.3645	0.3248

From Table 1, the Lyapunov index values of eight high and low frequency components are greater than zero, thus all of the components satisfy the chaos characteristics, and they can be predicted by establishing chaos model separately.

**Fig 4:**
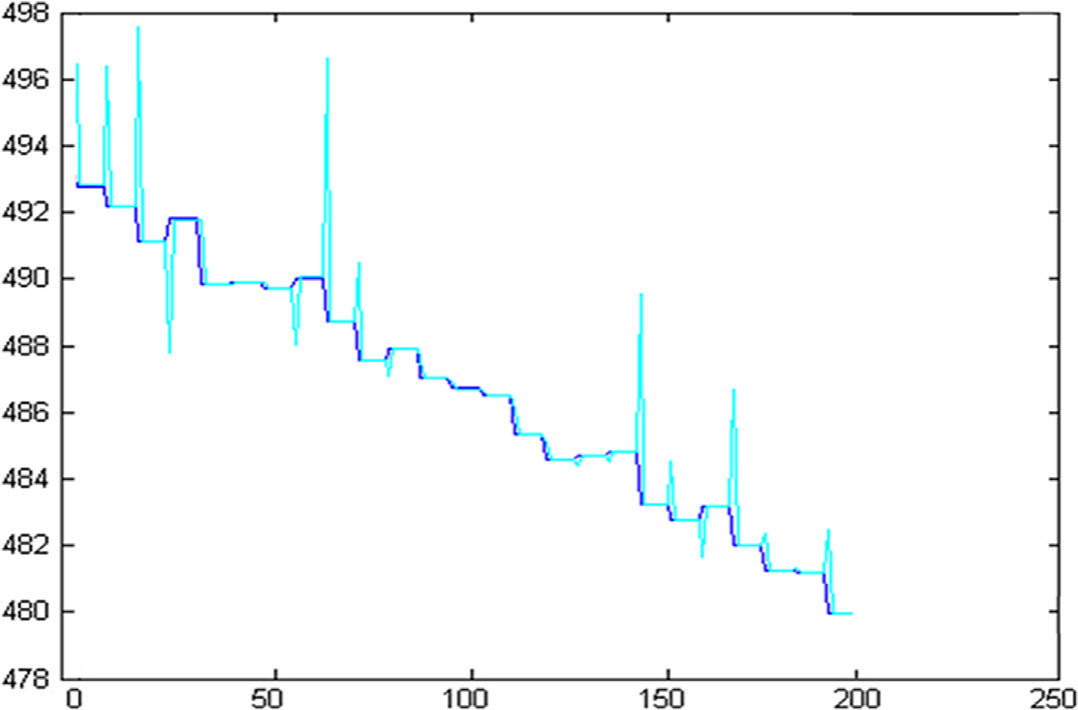
Prediction results of node (3, 0).

**Fig 5:**
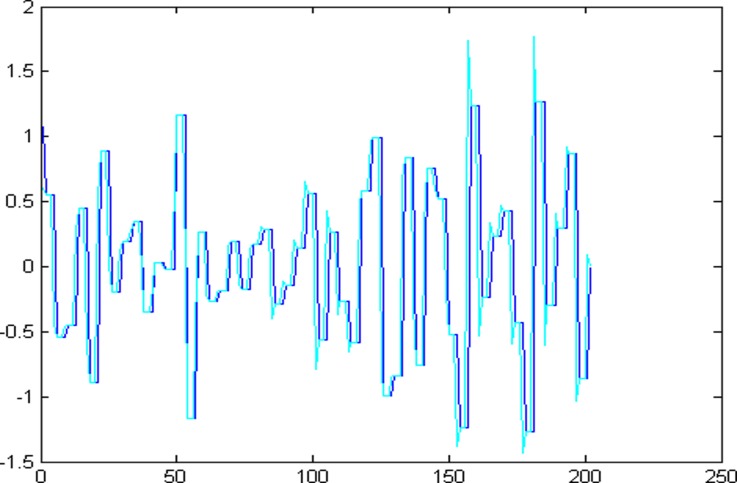
Prediction results of node (3, 1).

**Fig 6:**
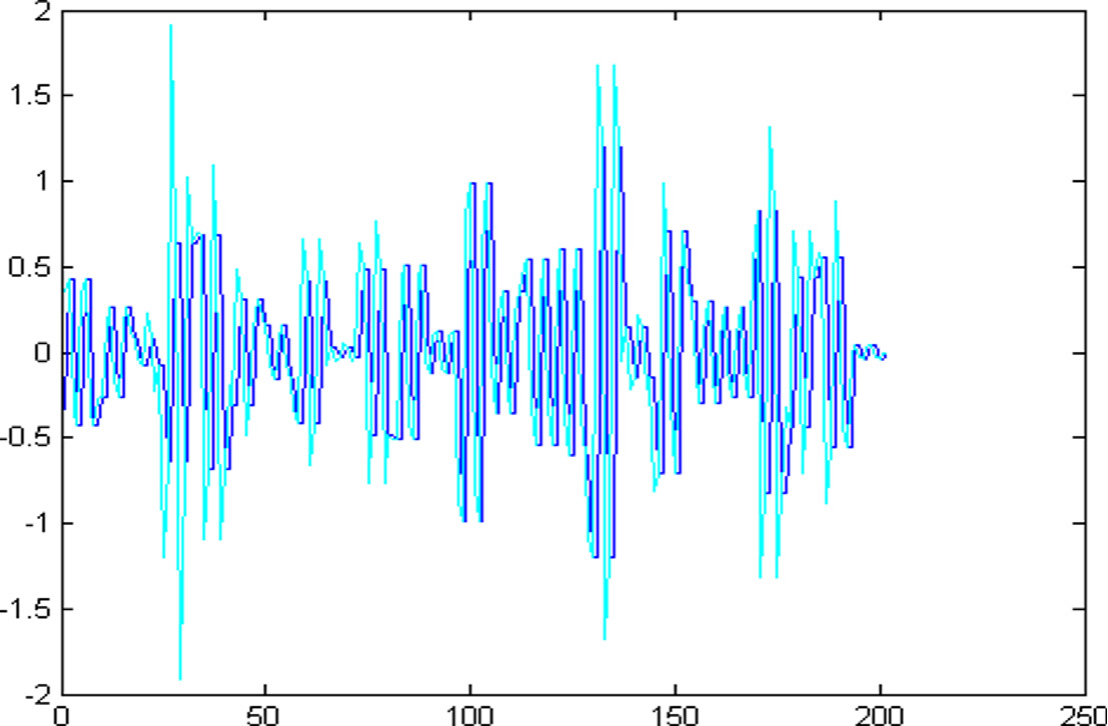
Prediction results of node (3, 2).

**Fig 7:**
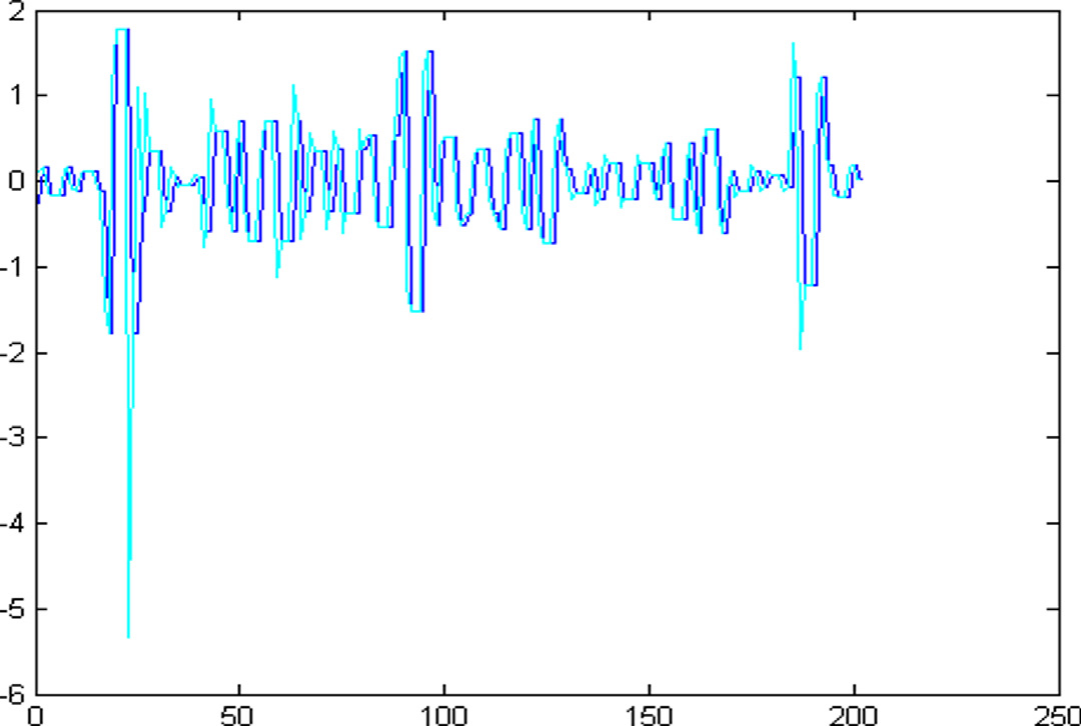
Prediction results of node (3, 3).

**Fig 8:**
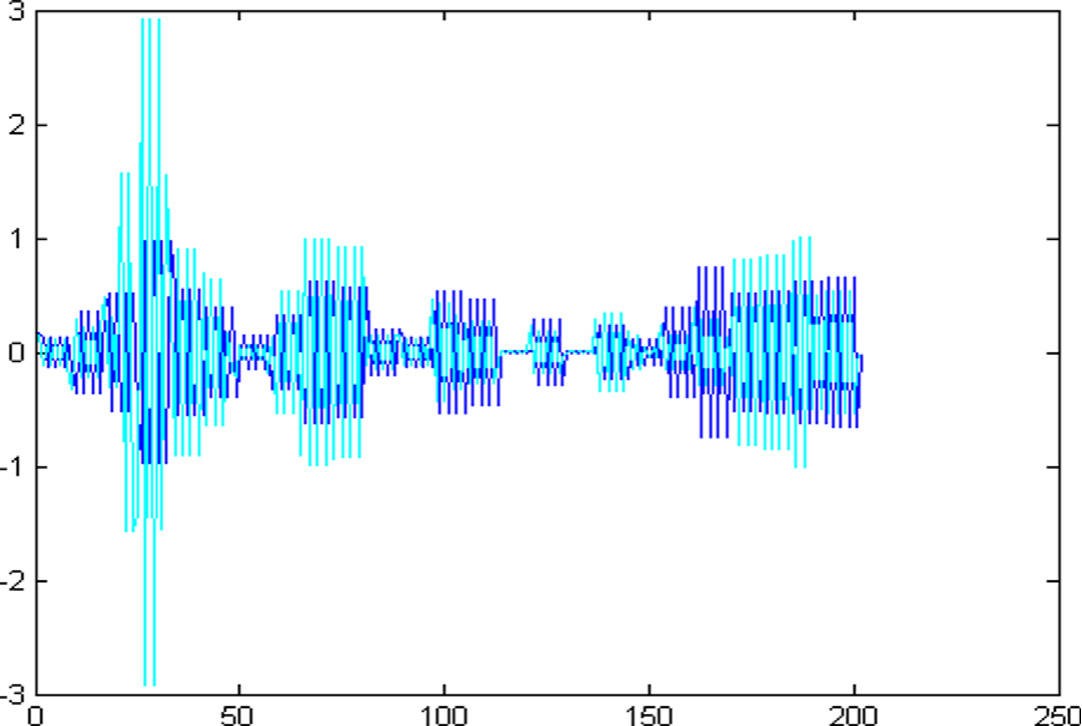
Prediction results of node (3, 4).

**Fig 9:**
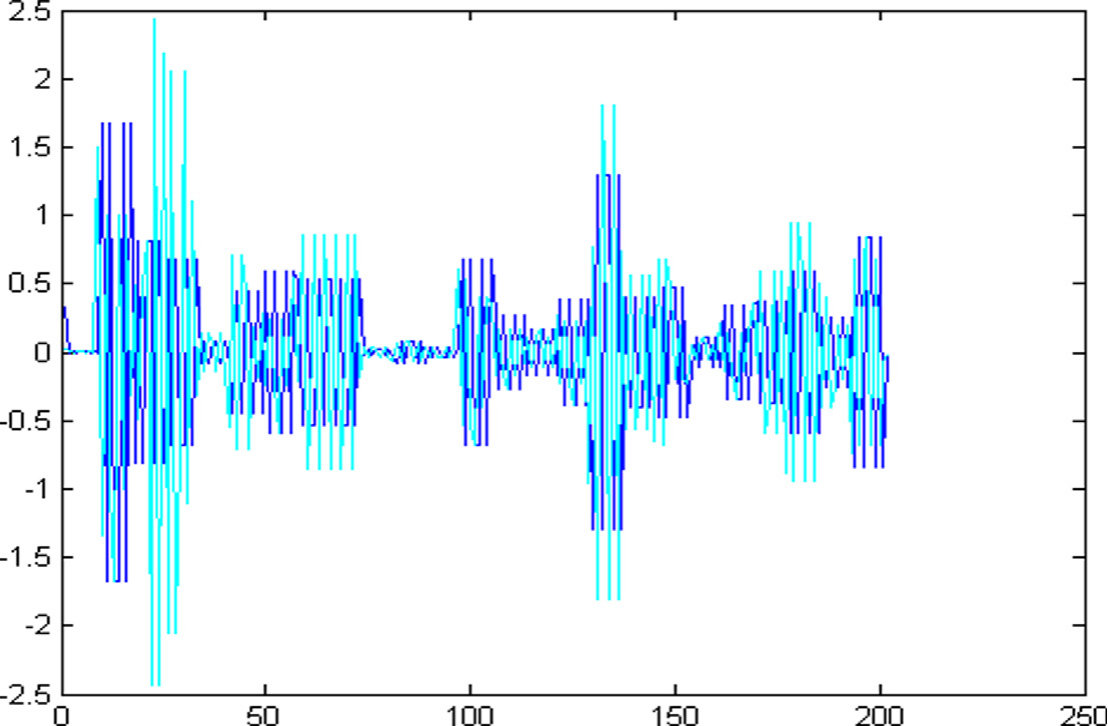
Prediction results of node (3, 5).

**Fig 10:**
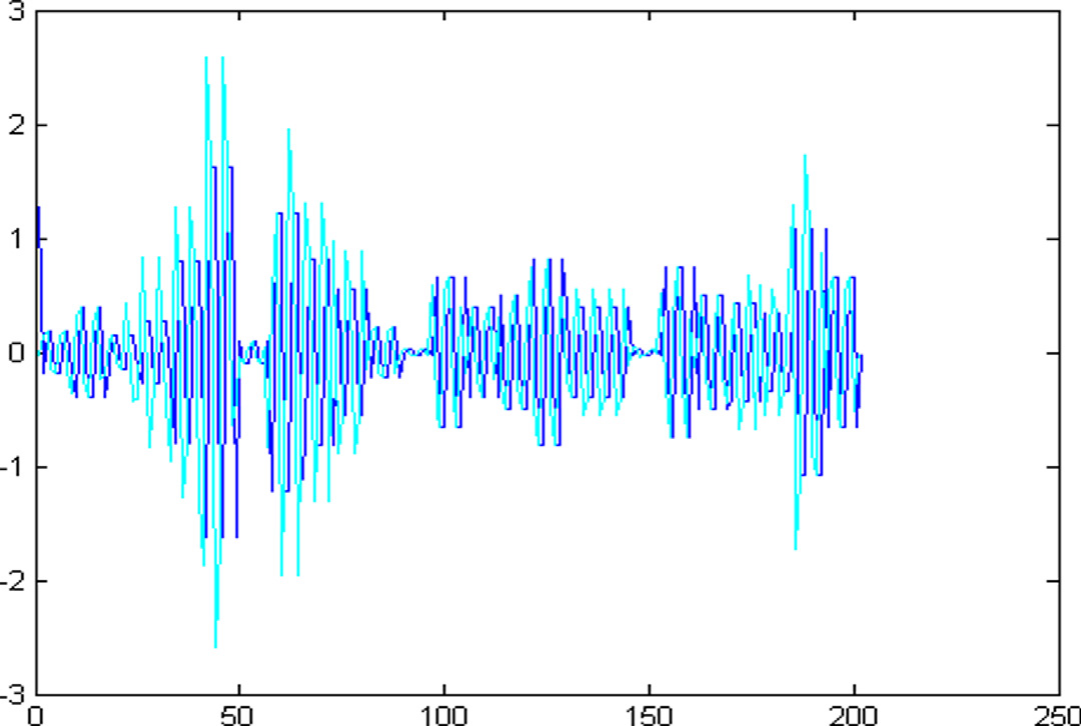
Prediction results of node (3, 6).

**Fig 11:**
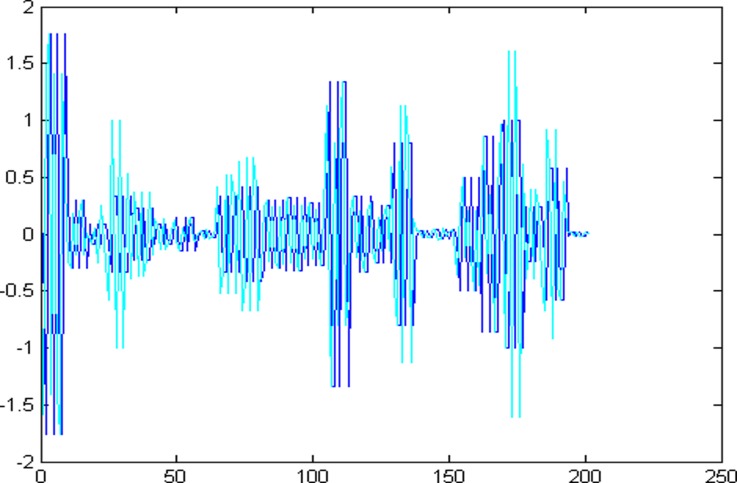
Prediction results of node (3, 7).

The 1001 super-path time series points can be divided into two parts, the first 799 data are training sample of training model and the other 202 data are test samples of the test model. First of all, establishing a chaotic model based on volterra series for 799 data points of eight high and low frequency components, then predict the data sequence of 202 data points. The forecast results are shown in Figs 4–11, where the navy blue line represents the actual value, and the light blue line represents the predicted value.

**Fig 12:**
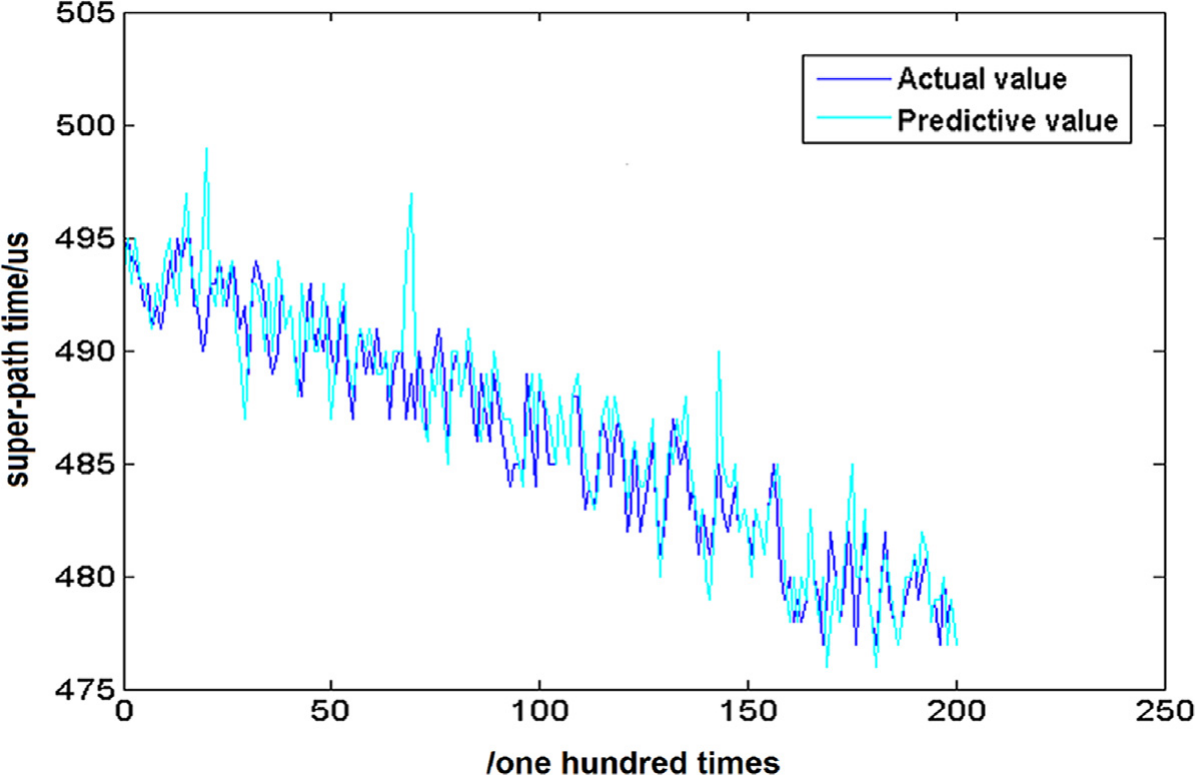
Comparison chart between prediction value and actual value.

The eight high and low frequency components are reconstructed by the wavelet packet reconstruction method, and the prediction results are compared with the 202 points of the original data, as shown in Fig 12.

**Fig 13:**
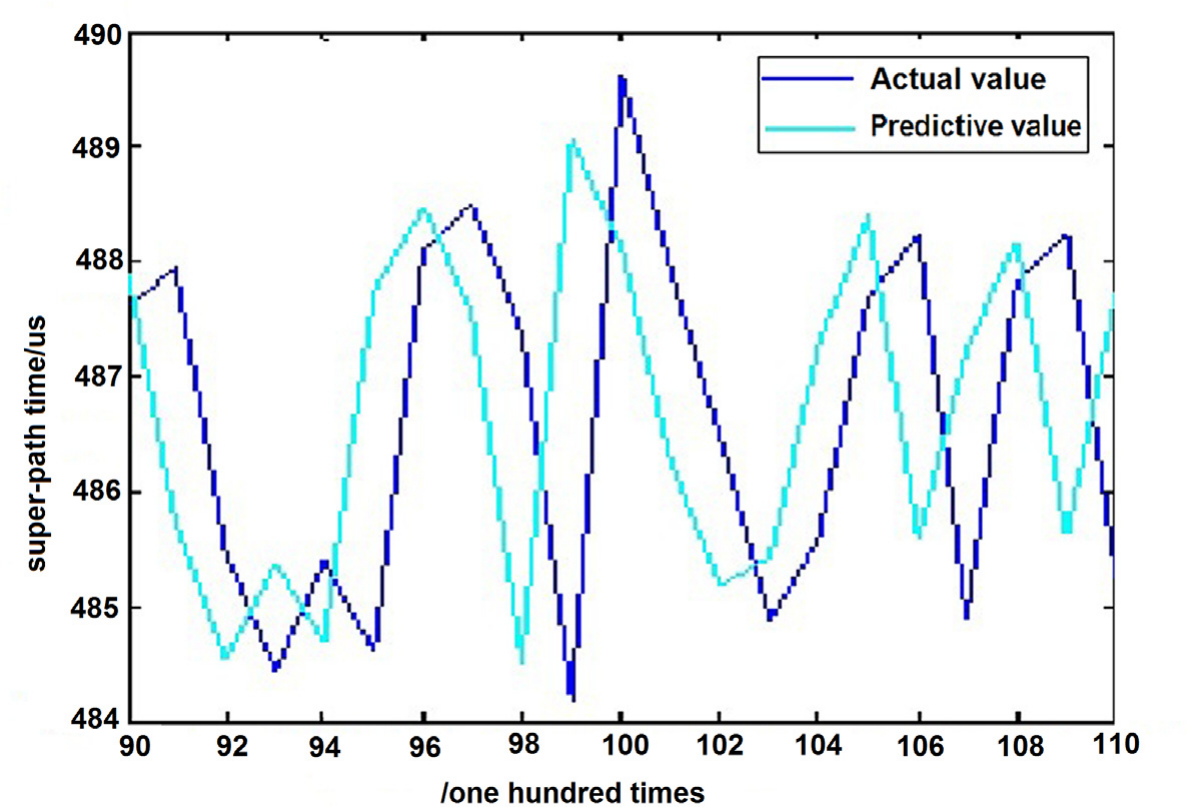
Partial discharge chart of prediction results.

In order to better observe the prediction results, the 20 data points cut from 90 to 110 have been enlarged partially in Fig 13. The amplification result is shown in Fig 13.

From the above predicted results, it can be seen that the prediction curve is very close to the real curve. The root mean square error *u* between the predicted results and the real values is 0.1881.

The value is extremely small, hence one can conclude that the chaotic forecasting model based on volterra series has an accurate forecasting capability.

## Conclusion

In this paper, an improved algorithm of wavelet packet-chaos model for life prediction of space relays based on volterra series is proposed. First, an improved cost function based on the Shannon information entropy and an improved best basis selection algorithm are proposed in the wavelet packet transform. Second, parameter optimization algorithm of SVS-LMS in volterra series is improved and the concept of entropy is integrated into the algorithm, the improved SVS-LMS algorithm has a faster speed and a better accuracy. An example shows that the improved algorithm of wavelet packet-chaos model based on volterra series can predict the life curve of the relay accurately and reflect the characteristics of the relay performance with sufficient accuracy, but there still remains an error between the prediction curve and the real curve, this requires us to make further improvements to the model.

## Supporting Information

S1 Appendix Super-path time series. (DOCX)
