# Supplementary material for: An Improved Wavelet Packet-Chaos Model for Life Prediction of Space Relays Based on Volterra Series
Source: PLoS One. 2016 Jun 29;11(6):e0158435. doi: 10.1371/journal.pone.0158435 (PMC4927151; doi:10.1371/journal.pone.0158435)
Supplement: S1 Appendix — (DOCX) [file pone.0158435.s001.docx]

| action times(/hundred times) | actual value of super- path time | action times(/hundred times) | prediction value of super-path time |
| --- | --- | --- | --- |
| 1 | 518 | 800 | 493 |
| 2 | 515 | 801 | 494 |
| 3 | 516 | 802 | 493 |
| 4 | 513 | 803 | 495 |
| 5 | 517 | 804 | 493 |
| 6 | 514 | 805 | 493 |
| 7 | 516 | 806 | 492 |
| 8 | 512 | 807 | 491 |
| 9 | 514 | 808 | 493 |
| 10 | 518 | 809 | 492 |
| 11 | 514 | 810 | 494 |
| 12 | 519 | 811 | 495 |
| 13 | 514 | 812 | 493 |
| 14 | 517 | 813 | 492 |
| 15 | 521 | 814 | 494 |
| 16 | 518 | 815 | 497 |
| 17 | 517 | 816 | 494 |
| 18 | 522 | 817 | 493 |
| 19 | 521 | 818 | 492 |
| 20 | 522 | 819 | 495 |
| 21 | 517 | 820 | 499 |
| 22 | 518 | 821 | 493 |
| 23 | 517 | 822 | 492 |
| 24 | 521 | 823 | 494 |
| 25 | 518 | 824 | 492 |
| 26 | 516 | 825 | 493 |
| 27 | 520 | 826 | 494 |
| 28 | 518 | 827 | 491 |
| 29 | 517 | 828 | 490 |
| 30 | 516 | 829 | 487 |
| 31 | 515 | 830 | 490 |
| 32 | 516 | 831 | 493 |
| 33 | 518 | 832 | 493 |
| 34 | 519 | 833 | 492 |
| 35 | 517 | 834 | 490 |
| 36 | 518 | 835 | 493 |
| 37 | 517 | 836 | 490 |
| 38 | 516 | 837 | 494 |
| 39 | 518 | 838 | 493 |
| 40 | 517 | 839 | 491 |
| 41 | 516 | 840 | 492 |
| 42 | 515 | 841 | 490 |
| 43 | 516 | 842 | 488 |
| 44 | 517 | 843 | 493 |
| 45 | 516 | 844 | 490 |
| 46 | 516 | 845 | 492 |
| 47 | 517 | 846 | 490 |
| 48 | 518 | 847 | 490 |
| 49 | 517 | 848 | 493 |
| 50 | 517 | 849 | 490 |
| 51 | 516 | 850 | 487 |
| 52 | 517 | 851 | 490 |
| 53 | 518 | 852 | 492 |
| 54 | 519 | 853 | 493 |
| 55 | 520 | 854 | 490 |
| 56 | 521 | 855 | 488 |
| 57 | 522 | 856 | 490 |
| 58 | 523 | 857 | 491 |
| 59 | 524 | 858 | 490 |
| 60 | 526 | 859 | 491 |
| 61 | 527 | 860 | 490 |
| 62 | 528 | 861 | 489 |
| 63 | 529 | 862 | 489 |
| 64 | 528 | 863 | 490 |
| 65 | 530 | 864 | 488 |
| 66 | 532 | 865 | 490 |
| 67 | 531 | 866 | 490 |
| 68 | 533 | 867 | 490 |
| 69 | 530 | 868 | 494 |
| 70 | 529 | 869 | 497 |
| 71 | 528 | 870 | 490 |
| 72 | 526 | 871 | 489 |
| 73 | 527 | 872 | 487 |
| 74 | 525 | 873 | 486 |
| 75 | 524 | 874 | 489 |
| 76 | 522 | 875 | 488 |
| 77 | 523 | 876 | 490 |
| 78 | 522 | 877 | 487 |
| 79 | 521 | 878 | 485 |
| 80 | 522 | 879 | 490 |
| 81 | 522 | 880 | 490 |
| 82 | 524 | 881 | 488 |
| 83 | 523 | 882 | 489 |
| 84 | 524 | 883 | 491 |
| 85 | 525 | 884 | 489 |
| 86 | 524 | 885 | 487 |
| 87 | 525 | 886 | 486 |
| 88 | 523 | 887 | 489 |
| 89 | 524 | 888 | 487 |
| 90 | 525 | 889 | 490 |
| 91 | 524 | 890 | 489 |
| 92 | 525 | 891 | 487 |
| 93 | 524 | 892 | 487 |
| 94 | 523 | 893 | 487 |
| 95 | 524 | 894 | 486 |
| 96 | 522 | 895 | 485 |
| 97 | 523 | 896 | 484 |
| 98 | 524 | 897 | 487 |
| 99 | 525 | 898 | 489 |
| 100 | 524 | 899 | 486 |
| 101 | 524 | 900 | 489 |
| 102 | 523 | 901 | 488 |
| 103 | 524 | 902 | 487 |
| 104 | 525 | 903 | 486 |
| 105 | 524 | 904 | 485 |
| 106 | 523 | 905 | 488 |
| 107 | 524 | 906 | 486 |
| 108 | 522 | 907 | 485 |
| 109 | 523 | 908 | 488 |
| 110 | 522 | 909 | 489 |
| 111 | 522 | 910 | 487 |
| 112 | 522 | 911 | 485 |
| 113 | 522 | 912 | 484 |
| 114 | 522 | 913 | 483 |
| 115 | 523 | 914 | 484 |
| 116 | 522 | 915 | 487 |
| 117 | 522 | 916 | 488 |
| 118 | 523 | 917 | 486 |
| 119 | 524 | 918 | 488 |
| 120 | 523 | 919 | 487 |
| 121 | 524 | 920 | 486 |
| 122 | 521 | 921 | 483 |
| 123 | 520 | 922 | 485 |
| 124 | 521 | 923 | 486 |
| 125 | 520 | 924 | 484 |
| 126 | 521 | 925 | 484 |
| 127 | 519 | 926 | 485 |
| 128 | 516 | 927 | 487 |
| 129 | 517 | 928 | 483 |
| 130 | 515 | 929 | 480 |
| 131 | 516 | 930 | 483 |
| 132 | 510 | 931 | 486 |
| 133 | 512 | 932 | 485 |
| 134 | 511 | 933 | 487 |
| 135 | 509 | 934 | 486 |
| 136 | 513 | 935 | 488 |
| 137 | 515 | 936 | 485 |
| 138 | 516 | 937 | 484 |
| 139 | 515 | 938 | 482 |
| 140 | 516 | 939 | 483 |
| 141 | 517 | 940 | 480 |
| 142 | 518 | 941 | 479 |
| 143 | 517 | 942 | 483 |
| 144 | 519 | 943 | 490 |
| 145 | 520 | 944 | 485 |
| 146 | 521 | 945 | 484 |
| 147 | 520 | 946 | 484 |
| 148 | 521 | 947 | 485 |
| 149 | 522 | 948 | 482 |
| 150 | 523 | 949 | 483 |
| 151 | 522 | 950 | 482 |
| 152 | 521 | 951 | 480 |
| 153 | 523 | 952 | 483 |
| 154 | 524 | 953 | 482 |
| 155 | 523 | 954 | 481 |
| 156 | 522 | 955 | 483 |
| 157 | 523 | 956 | 484 |
| 158 | 521 | 957 | 485 |
| 159 | 520 | 958 | 483 |
| 160 | 521 | 959 | 480 |
| 161 | 520 | 960 | 478 |
| 162 | 521 | 961 | 480 |
| 163 | 520 | 962 | 478 |
| 164 | 521 | 963 | 480 |
| 165 | 520 | 964 | 479 |
| 166 | 521 | 965 | 483 |
| 167 | 519 | 966 | 480 |
| 168 | 521 | 967 | 478 |
| 169 | 520 | 968 | 480 |
| 170 | 521 | 969 | 476 |
| 171 | 519 | 970 | 478 |
| 172 | 520 | 971 | 480 |
| 173 | 519 | 972 | 478 |
| 174 | 521 | 973 | 480 |
| 175 | 519 | 974 | 483 |
| 176 | 521 | 975 | 485 |
| 177 | 520 | 976 | 480 |
| 178 | 518 | 977 | 480 |
| 179 | 519 | 978 | 483 |
| 180 | 520 | 979 | 479 |
| 181 | 519 | 980 | 478 |
| 182 | 518 | 981 | 476 |
| 183 | 520 | 982 | 480 |
| 184 | 519 | 983 | 481 |
| 185 | 518 | 984 | 480 |
| 186 | 519 | 985 | 478 |
| 187 | 517 | 986 | 477 |
| 188 | 518 | 987 | 478 |
| 189 | 517 | 988 | 480 |
| 190 | 518 | 989 | 480 |
| 191 | 519 | 990 | 481 |
| 192 | 517 | 991 | 480 |
| 193 | 518 | 992 | 482 |
| 194 | 517 | 993 | 481 |
| 195 | 518 | 994 | 478 |
| 196 | 517 | 995 | 479 |
| 197 | 518 | 996 | 479 |
| 198 | 518 | 997 | 480 |
| 199 | 519 | 998 | 477 |
| 200 | 520 | 999 | 479 |
| 201 | 518 | 1000 | 478 |
| 202 | 517 | 1001 | 477 |
| 203 | 518 |  |  |
| 204 | 519 |  |  |
| 205 | 518 |  |  |
| 206 | 518 |  |  |
| 207 | 519 |  |  |
| 208 | 518 |  |  |
| 209 | 517 |  |  |
| 210 | 516 |  |  |
| 211 | 517 |  |  |
| 212 | 517 |  |  |
| 213 | 518 |  |  |
| 214 | 516 |  |  |
| 215 | 516 |  |  |
| 216 | 517 |  |  |
| 217 | 517 |  |  |
| 218 | 516 |  |  |
| 219 | 517 |  |  |
| 220 | 516 |  |  |
| 221 | 517 |  |  |
| 222 | 515 |  |  |
| 223 | 514 |  |  |
| 224 | 513 |  |  |
| 225 | 514 |  |  |
| 226 | 512 |  |  |
| 227 | 511 |  |  |
| 228 | 510 |  |  |
| 229 | 509 |  |  |
| 230 | 507 |  |  |
| 231 | 508 |  |  |
| 232 | 507 |  |  |
| 233 | 506 |  |  |
| 234 | 507 |  |  |
| 235 | 506 |  |  |
| 236 | 508 |  |  |
| 237 | 506 |  |  |
| 238 | 507 |  |  |
| 239 | 506 |  |  |
| 240 | 505 |  |  |
| 241 | 505 |  |  |
| 242 | 507 |  |  |
| 243 | 510 |  |  |
| 244 | 509 |  |  |
| 245 | 508 |  |  |
| 246 | 512 |  |  |
| 247 | 510 |  |  |
| 248 | 514 |  |  |
| 249 | 515 |  |  |
| 250 | 518 |  |  |
| 251 | 519 |  |  |
| 252 | 520 |  |  |
| 253 | 519 |  |  |
| 254 | 523 |  |  |
| 255 | 524 |  |  |
| 256 | 525 |  |  |
| 257 | 526 |  |  |
| 258 | 527 |  |  |
| 259 | 529 |  |  |
| 260 | 528 |  |  |
| 261 | 529 |  |  |
| 262 | 530 |  |  |
| 263 | 533 |  |  |
| 264 | 534 |  |  |
| 265 | 533 |  |  |
| 266 | 534 |  |  |
| 267 | 535 |  |  |
| 268 | 534 |  |  |
| 269 | 535 |  |  |
| 270 | 534 |  |  |
| 271 | 530 |  |  |
| 272 | 531 |  |  |
| 273 | 530 |  |  |
| 274 | 531 |  |  |
| 275 | 529 |  |  |
| 276 | 527 |  |  |
| 277 | 527 |  |  |
| 278 | 526 |  |  |
| 279 | 520 |  |  |
| 280 | 519 |  |  |
| 281 | 517 |  |  |
| 282 | 518 |  |  |
| 283 | 517 |  |  |
| 284 | 519 |  |  |
| 285 | 520 |  |  |
| 286 | 523 |  |  |
| 287 | 524 |  |  |
| 288 | 526 |  |  |
| 289 | 529 |  |  |
| 290 | 530 |  |  |
| 291 | 531 |  |  |
| 292 | 530 |  |  |
| 293 | 531 |  |  |
| 294 | 533 |  |  |
| 295 | 535 |  |  |
| 296 | 536 |  |  |
| 297 | 535 |  |  |
| 298 | 536 |  |  |
| 299 | 537 |  |  |
| 300 | 538 |  |  |
| 301 | 540 |  |  |
| 302 | 540 |  |  |
| 303 | 539 |  |  |
| 304 | 537 |  |  |
| 305 | 535 |  |  |
| 306 | 533 |  |  |
| 307 | 531 |  |  |
| 308 | 530 |  |  |
| 309 | 528 |  |  |
| 310 | 525 |  |  |
| 311 | 523 |  |  |
| 312 | 520 |  |  |
| 313 | 520 |  |  |
| 314 | 518 |  |  |
| 315 | 516 |  |  |
| 316 | 516 |  |  |
| 317 | 517 |  |  |
| 318 | 516 |  |  |
| 319 | 515 |  |  |
| 320 | 516 |  |  |
| 321 | 515 |  |  |
| 322 | 514 |  |  |
| 323 | 515 |  |  |
| 324 | 514 |  |  |
| 325 | 514 |  |  |
| 326 | 515 |  |  |
| 327 | 515 |  |  |
| 328 | 514 |  |  |
| 329 | 515 |  |  |
| 330 | 515 |  |  |
| 331 | 514 |  |  |
| 332 | 515 |  |  |
| 333 | 514 |  |  |
| 334 | 513 |  |  |
| 335 | 514 |  |  |
| 336 | 514 |  |  |
| 337 | 513 |  |  |
| 338 | 515 |  |  |
| 339 | 513 |  |  |
| 340 | 514 |  |  |
| 341 | 514 |  |  |
| 342 | 514 |  |  |
| 343 | 513 |  |  |
| 344 | 515 |  |  |
| 345 | 514 |  |  |
| 346 | 514 |  |  |
| 347 | 515 |  |  |
| 348 | 514 |  |  |
| 349 | 515 |  |  |
| 350 | 514 |  |  |
| 351 | 513 |  |  |
| 352 | 514 |  |  |
| 353 | 514 |  |  |
| 354 | 515 |  |  |
| 355 | 513 |  |  |
| 356 | 514 |  |  |
| 357 | 514 |  |  |
| 358 | 514 |  |  |
| 359 | 513 |  |  |
| 360 | 512 |  |  |
| 361 | 513 |  |  |
| 362 | 513 |  |  |
| 363 | 513 |  |  |
| 364 | 512 |  |  |
| 365 | 513 |  |  |
| 366 | 513 |  |  |
| 367 | 513 |  |  |
| 368 | 512 |  |  |
| 369 | 512 |  |  |
| 370 | 513 |  |  |
| 371 | 515 |  |  |
| 372 | 517 |  |  |
| 373 | 518 |  |  |
| 374 | 519 |  |  |
| 375 | 518 |  |  |
| 376 | 520 |  |  |
| 377 | 524 |  |  |
| 378 | 523 |  |  |
| 379 | 525 |  |  |
| 380 | 527 |  |  |
| 381 | 528 |  |  |
| 382 | 529 |  |  |
| 383 | 530 |  |  |
| 384 | 529 |  |  |
| 385 | 527 |  |  |
| 386 | 526 |  |  |
| 387 | 527 |  |  |
| 388 | 525 |  |  |
| 389 | 524 |  |  |
| 390 | 525 |  |  |
| 391 | 523 |  |  |
| 392 | 520 |  |  |
| 393 | 518 |  |  |
| 394 | 517 |  |  |
| 395 | 518 |  |  |
| 396 | 515 |  |  |
| 397 | 513 |  |  |
| 398 | 514 |  |  |
| 399 | 513 |  |  |
| 400 | 510 |  |  |
| 401 | 507 |  |  |
| 402 | 505 |  |  |
| 403 | 504 |  |  |
| 404 | 503 |  |  |
| 405 | 501 |  |  |
| 406 | 500 |  |  |
| 407 | 503 |  |  |
| 408 | 505 |  |  |
| 409 | 509 |  |  |
| 410 | 507 |  |  |
| 411 | 510 |  |  |
| 412 | 514 |  |  |
| 413 | 513 |  |  |
| 414 | 512 |  |  |
| 415 | 513 |  |  |
| 416 | 514 |  |  |
| 417 | 512 |  |  |
| 418 | 512 |  |  |
| 419 | 513 |  |  |
| 420 | 512 |  |  |
| 421 | 513 |  |  |
| 422 | 512 |  |  |
| 423 | 512 |  |  |
| 424 | 511 |  |  |
| 425 | 512 |  |  |
| 426 | 511 |  |  |
| 427 | 512 |  |  |
| 428 | 511 |  |  |
| 429 | 511 |  |  |
| 430 | 512 |  |  |
| 431 | 511 |  |  |
| 432 | 511 |  |  |
| 433 | 512 |  |  |
| 434 | 510 |  |  |
| 435 | 511 |  |  |
| 436 | 510 |  |  |
| 437 | 512 |  |  |
| 438 | 510 |  |  |
| 439 | 509 |  |  |
| 440 | 508 |  |  |
| 441 | 509 |  |  |
| 442 | 510 |  |  |
| 443 | 508 |  |  |
| 444 | 508 |  |  |
| 445 | 507 |  |  |
| 446 | 508 |  |  |
| 447 | 507 |  |  |
| 448 | 507 |  |  |
| 449 | 506 |  |  |
| 450 | 507 |  |  |
| 451 | 506 |  |  |
| 452 | 506 |  |  |
| 453 | 507 |  |  |
| 454 | 507 |  |  |
| 455 | 505 |  |  |
| 456 | 504 |  |  |
| 457 | 505 |  |  |
| 458 | 504 |  |  |
| 459 | 505 |  |  |
| 460 | 506 |  |  |
| 461 | 507 |  |  |
| 462 | 506 |  |  |
| 463 | 507 |  |  |
| 464 | 507 |  |  |
| 465 | 508 |  |  |
| 466 | 507 |  |  |
| 467 | 508 |  |  |
| 468 | 509 |  |  |
| 469 | 509 |  |  |
| 470 | 510 |  |  |
| 471 | 512 |  |  |
| 472 | 512 |  |  |
| 473 | 511 |  |  |
| 474 | 510 |  |  |
| 475 | 510 |  |  |
| 476 | 511 |  |  |
| 477 | 509 |  |  |
| 478 | 510 |  |  |
| 479 | 511 |  |  |
| 480 | 510 |  |  |
| 481 | 509 |  |  |
| 482 | 510 |  |  |
| 483 | 510 |  |  |
| 484 | 509 |  |  |
| 485 | 508 |  |  |
| 486 | 510 |  |  |
| 487 | 508 |  |  |
| 488 | 509 |  |  |
| 489 | 509 |  |  |
| 490 | 510 |  |  |
| 491 | 509 |  |  |
| 492 | 509 |  |  |
| 493 | 509 |  |  |
| 494 | 510 |  |  |
| 495 | 510 |  |  |
| 496 | 509 |  |  |
| 497 | 510 |  |  |
| 498 | 510 |  |  |
| 499 | 511 |  |  |
| 500 | 511 |  |  |
| 501 | 510 |  |  |
| 502 | 511 |  |  |
| 503 | 513 |  |  |
| 504 | 515 |  |  |
| 505 | 517 |  |  |
| 506 | 520 |  |  |
| 507 | 523 |  |  |
| 508 | 525 |  |  |
| 509 | 527 |  |  |
| 510 | 528 |  |  |
| 511 | 528 |  |  |
| 512 | 525 |  |  |
| 513 | 523 |  |  |
| 514 | 520 |  |  |
| 515 | 519 |  |  |
| 516 | 515 |  |  |
| 517 | 513 |  |  |
| 518 | 510 |  |  |
| 519 | 509 |  |  |
| 520 | 510 |  |  |
| 521 | 509 |  |  |
| 522 | 510 |  |  |
| 523 | 509 |  |  |
| 524 | 510 |  |  |
| 525 | 511 |  |  |
| 526 | 509 |  |  |
| 527 | 508 |  |  |
| 528 | 509 |  |  |
| 529 | 508 |  |  |
| 530 | 507 |  |  |
| 531 | 508 |  |  |
| 532 | 509 |  |  |
| 533 | 507 |  |  |
| 534 | 508 |  |  |
| 535 | 507 |  |  |
| 536 | 507 |  |  |
| 537 | 508 |  |  |
| 538 | 505 |  |  |
| 539 | 503 |  |  |
| 540 | 500 |  |  |
| 541 | 499 |  |  |
| 542 | 498 |  |  |
| 543 | 506 |  |  |
| 544 | 508 |  |  |
| 545 | 509 |  |  |
| 546 | 509 |  |  |
| 547 | 509 |  |  |
| 548 | 508 |  |  |
| 549 | 509 |  |  |
| 550 | 508 |  |  |
| 551 | 507 |  |  |
| 552 | 508 |  |  |
| 553 | 506 |  |  |
| 554 | 507 |  |  |
| 555 | 508 |  |  |
| 556 | 506 |  |  |
| 557 | 506 |  |  |
| 558 | 507 |  |  |
| 559 | 506 |  |  |
| 560 | 507 |  |  |
| 561 | 506 |  |  |
| 562 | 505 |  |  |
| 563 | 506 |  |  |
| 564 | 504 |  |  |
| 565 | 503 |  |  |
| 566 | 504 |  |  |
| 567 | 504 |  |  |
| 568 | 503 |  |  |
| 569 | 504 |  |  |
| 570 | 503 |  |  |
| 571 | 505 |  |  |
| 572 | 506 |  |  |
| 573 | 505 |  |  |
| 574 | 507 |  |  |
| 575 | 506 |  |  |
| 576 | 506 |  |  |
| 577 | 507 |  |  |
| 578 | 509 |  |  |
| 579 | 510 |  |  |
| 580 | 515 |  |  |
| 581 | 514 |  |  |
| 582 | 518 |  |  |
| 583 | 517 |  |  |
| 584 | 516 |  |  |
| 585 | 520 |  |  |
| 586 | 525 |  |  |
| 587 | 520 |  |  |
| 588 | 521 |  |  |
| 589 | 519 |  |  |
| 590 | 512 |  |  |
| 591 | 510 |  |  |
| 592 | 511 |  |  |
| 593 | 510 |  |  |
| 594 | 506 |  |  |
| 595 | 507 |  |  |
| 596 | 506 |  |  |
| 597 | 505 |  |  |
| 598 | 506 |  |  |
| 599 | 507 |  |  |
| 600 | 506 |  |  |
| 601 | 504 |  |  |
| 602 | 505 |  |  |
| 603 | 507 |  |  |
| 604 | 506 |  |  |
| 605 | 505 |  |  |
| 606 | 504 |  |  |
| 607 | 504 |  |  |
| 608 | 505 |  |  |
| 609 | 504 |  |  |
| 610 | 503 |  |  |
| 611 | 504 |  |  |
| 612 | 505 |  |  |
| 613 | 504 |  |  |
| 614 | 506 |  |  |
| 615 | 507 |  |  |
| 616 | 509 |  |  |
| 617 | 508 |  |  |
| 618 | 507 |  |  |
| 619 | 508 |  |  |
| 620 | 507 |  |  |
| 621 | 506 |  |  |
| 622 | 505 |  |  |
| 623 | 504 |  |  |
| 624 | 507 |  |  |
| 625 | 506 |  |  |
| 626 | 507 |  |  |
| 627 | 508 |  |  |
| 628 | 509 |  |  |
| 629 | 510 |  |  |
| 630 | 515 |  |  |
| 631 | 510 |  |  |
| 632 | 506 |  |  |
| 633 | 504 |  |  |
| 634 | 504 |  |  |
| 635 | 503 |  |  |
| 636 | 503 |  |  |
| 637 | 504 |  |  |
| 638 | 502 |  |  |
| 639 | 504 |  |  |
| 640 | 505 |  |  |
| 641 | 503 |  |  |
| 642 | 504 |  |  |
| 643 | 502 |  |  |
| 644 | 502 |  |  |
| 645 | 501 |  |  |
| 646 | 503 |  |  |
| 647 | 504 |  |  |
| 648 | 501 |  |  |
| 649 | 503 |  |  |
| 650 | 504 |  |  |
| 651 | 500 |  |  |
| 652 | 497 |  |  |
| 653 | 498 |  |  |
| 654 | 496 |  |  |
| 655 | 495 |  |  |
| 656 | 493 |  |  |
| 657 | 494 |  |  |
| 658 | 490 |  |  |
| 659 | 492 |  |  |
| 660 | 497 |  |  |
| 661 | 496 |  |  |
| 662 | 498 |  |  |
| 663 | 497 |  |  |
| 664 | 499 |  |  |
| 665 | 503 |  |  |
| 666 | 502 |  |  |
| 667 | 501 |  |  |
| 668 | 500 |  |  |
| 669 | 502 |  |  |
| 670 | 501 |  |  |
| 671 | 503 |  |  |
| 672 | 504 |  |  |
| 673 | 501 |  |  |
| 674 | 500 |  |  |
| 675 | 502 |  |  |
| 676 | 501 |  |  |
| 677 | 502 |  |  |
| 678 | 503 |  |  |
| 679 | 504 |  |  |
| 680 | 503 |  |  |
| 681 | 501 |  |  |
| 682 | 500 |  |  |
| 683 | 501 |  |  |
| 684 | 503 |  |  |
| 685 | 502 |  |  |
| 686 | 504 |  |  |
| 687 | 501 |  |  |
| 688 | 500 |  |  |
| 689 | 499 |  |  |
| 690 | 500 |  |  |
| 691 | 501 |  |  |
| 692 | 502 |  |  |
| 693 | 503 |  |  |
| 694 | 502 |  |  |
| 695 | 503 |  |  |
| 696 | 501 |  |  |
| 697 | 503 |  |  |
| 698 | 501 |  |  |
| 699 | 502 |  |  |
| 700 | 503 |  |  |
| 701 | 505 |  |  |
| 702 | 504 |  |  |
| 703 | 506 |  |  |
| 704 | 507 |  |  |
| 705 | 510 |  |  |
| 706 | 509 |  |  |
| 707 | 512 |  |  |
| 708 | 511 |  |  |
| 709 | 507 |  |  |
| 710 | 508 |  |  |
| 711 | 506 |  |  |
| 712 | 507 |  |  |
| 713 | 504 |  |  |
| 714 | 500 |  |  |
| 715 | 498 |  |  |
| 716 | 499 |  |  |
| 717 | 497 |  |  |
| 718 | 498 |  |  |
| 719 | 499 |  |  |
| 720 | 498 |  |  |
| 721 | 496 |  |  |
| 722 | 497 |  |  |
| 723 | 496 |  |  |
| 724 | 495 |  |  |
| 725 | 498 |  |  |
| 726 | 499 |  |  |
| 727 | 497 |  |  |
| 728 | 496 |  |  |
| 729 | 497 |  |  |
| 730 | 495 |  |  |
| 731 | 497 |  |  |
| 732 | 496 |  |  |
| 733 | 497 |  |  |
| 734 | 498 |  |  |
| 735 | 496 |  |  |
| 736 | 495 |  |  |
| 737 | 498 |  |  |
| 738 | 495 |  |  |
| 739 | 497 |  |  |
| 740 | 495 |  |  |
| 741 | 497 |  |  |
| 742 | 496 |  |  |
| 743 | 500 |  |  |
| 744 | 509 |  |  |
| 745 | 503 |  |  |
| 746 | 500 |  |  |
| 747 | 501 |  |  |
| 748 | 499 |  |  |
| 749 | 495 |  |  |
| 750 | 496 |  |  |
| 751 | 495 |  |  |
| 752 | 493 |  |  |
| 753 | 494 |  |  |
| 754 | 493 |  |  |
| 755 | 495 |  |  |
| 756 | 494 |  |  |
| 757 | 496 |  |  |
| 758 | 495 |  |  |
| 759 | 493 |  |  |
| 760 | 495 |  |  |
| 761 | 494 |  |  |
| 762 | 493 |  |  |
| 763 | 495 |  |  |
| 764 | 493 |  |  |
| 765 | 494 |  |  |
| 766 | 496 |  |  |
| 767 | 493 |  |  |
| 768 | 494 |  |  |
| 769 | 497 |  |  |
| 770 | 495 |  |  |
| 771 | 493 |  |  |
| 772 | 493 |  |  |
| 773 | 492 |  |  |
| 774 | 494 |  |  |
| 775 | 492 |  |  |
| 776 | 491 |  |  |
| 777 | 492 |  |  |
| 778 | 490 |  |  |
| 779 | 492 |  |  |
| 780 | 493 |  |  |
| 781 | 491 |  |  |
| 782 | 490 |  |  |
| 783 | 492 |  |  |
| 784 | 494 |  |  |
| 785 | 493 |  |  |
| 786 | 492 |  |  |
| 787 | 495 |  |  |
| 788 | 493 |  |  |
| 789 | 499 |  |  |
| 790 | 500 |  |  |
| 791 | 505 |  |  |
| 792 | 502 |  |  |
| 793 | 500 |  |  |
| 794 | 496 |  |  |
| 795 | 497 |  |  |
| 796 | 496 |  |  |
| 797 | 495 |  |  |
| 798 | 496 |  |  |
| 799 | 495 |  |  |
| 800 | 494 |  |  |
| 801 | 495 |  |  |
| 802 | 494 |  |  |
| 803 | 494 |  |  |
| 804 | 493 |  |  |
| 805 | 492 |  |  |
| 806 | 493 |  |  |
| 807 | 491 |  |  |
| 808 | 492 |  |  |
| 809 | 491 |  |  |
| 810 | 492 |  |  |
| 811 | 494 |  |  |
| 812 | 493 |  |  |
| 813 | 495 |  |  |
| 814 | 494 |  |  |
| 815 | 495 |  |  |
| 816 | 495 |  |  |
| 817 | 492 |  |  |
| 818 | 492 |  |  |
| 819 | 490 |  |  |
| 820 | 491 |  |  |
| 821 | 493 |  |  |
| 822 | 493 |  |  |
| 823 | 494 |  |  |
| 824 | 493 |  |  |
| 825 | 492 |  |  |
| 826 | 494 |  |  |
| 827 | 493 |  |  |
| 828 | 491 |  |  |
| 829 | 492 |  |  |
| 830 | 489 |  |  |
| 831 | 493 |  |  |
| 832 | 494 |  |  |
| 833 | 493 |  |  |
| 834 | 492 |  |  |
| 835 | 490 |  |  |
| 836 | 489 |  |  |
| 837 | 490 |  |  |
| 838 | 493 |  |  |
| 839 | 491 |  |  |
| 840 | 492 |  |  |
| 841 | 490 |  |  |
| 842 | 489 |  |  |
| 843 | 488 |  |  |
| 844 | 492 |  |  |
| 845 | 493 |  |  |
| 846 | 490 |  |  |
| 847 | 491 |  |  |
| 848 | 490 |  |  |
| 849 | 492 |  |  |
| 850 | 490 |  |  |
| 851 | 489 |  |  |
| 852 | 491 |  |  |
| 853 | 492 |  |  |
| 854 | 489 |  |  |
| 855 | 487 |  |  |
| 856 | 490 |  |  |
| 857 | 491 |  |  |
| 858 | 489 |  |  |
| 859 | 490 |  |  |
| 860 | 489 |  |  |
| 861 | 491 |  |  |
| 862 | 489 |  |  |
| 863 | 490 |  |  |
| 864 | 487 |  |  |
| 865 | 489 |  |  |
| 866 | 490 |  |  |
| 867 | 490 |  |  |
| 868 | 487 |  |  |
| 869 | 489 |  |  |
| 870 | 487 |  |  |
| 871 | 490 |  |  |
| 872 | 489 |  |  |
| 873 | 486 |  |  |
| 874 | 489 |  |  |
| 875 | 490 |  |  |
| 876 | 491 |  |  |
| 877 | 489 |  |  |
| 878 | 486 |  |  |
| 879 | 489 |  |  |
| 880 | 490 |  |  |
| 881 | 488 |  |  |
| 882 | 489 |  |  |
| 883 | 490 |  |  |
| 884 | 487 |  |  |
| 885 | 486 |  |  |
| 886 | 489 |  |  |
| 887 | 487 |  |  |
| 888 | 486 |  |  |
| 889 | 489 |  |  |
| 890 | 488 |  |  |
| 891 | 486 |  |  |
| 892 | 485 |  |  |
| 893 | 484 |  |  |
| 894 | 485 |  |  |
| 895 | 485 |  |  |
| 896 | 484 |  |  |
| 897 | 489 |  |  |
| 898 | 486 |  |  |
| 899 | 484 |  |  |
| 900 | 489 |  |  |
| 901 | 488 |  |  |
| 902 | 485 |  |  |
| 903 | 485 |  |  |
| 904 | 485 |  |  |
| 905 | 488 |  |  |
| 906 | 486 |  |  |
| 907 | 485 |  |  |
| 908 | 488 |  |  |
| 909 | 488 |  |  |
| 910 | 485 |  |  |
| 911 | 483 |  |  |
| 912 | 484 |  |  |
| 913 | 483 |  |  |
| 914 | 485 |  |  |
| 915 | 487 |  |  |
| 916 | 486 |  |  |
| 917 | 484 |  |  |
| 918 | 486 |  |  |
| 919 | 487 |  |  |
| 920 | 485 |  |  |
| 921 | 482 |  |  |
| 922 | 483 |  |  |
| 923 | 486 |  |  |
| 924 | 482 |  |  |
| 925 | 483 |  |  |
| 926 | 484 |  |  |
| 927 | 486 |  |  |
| 928 | 483 |  |  |
| 929 | 481 |  |  |
| 930 | 482 |  |  |
| 931 | 485 |  |  |
| 932 | 487 |  |  |
| 933 | 486 |  |  |
| 934 | 485 |  |  |
| 935 | 486 |  |  |
| 936 | 483 |  |  |
| 937 | 484 |  |  |
| 938 | 481 |  |  |
| 939 | 483 |  |  |
| 940 | 482 |  |  |
| 941 | 481 |  |  |
| 942 | 483 |  |  |
| 943 | 485 |  |  |
| 944 | 483 |  |  |
| 945 | 482 |  |  |
| 946 | 483 |  |  |
| 947 | 484 |  |  |
| 948 | 482 |  |  |
| 949 | 483 |  |  |
| 950 | 482 |  |  |
| 951 | 481 |  |  |
| 952 | 483 |  |  |
| 953 | 482 |  |  |
| 954 | 481 |  |  |
| 955 | 483 |  |  |
| 956 | 485 |  |  |
| 957 | 483 |  |  |
| 958 | 480 |  |  |
| 959 | 479 |  |  |
| 960 | 480 |  |  |
| 961 | 478 |  |  |
| 962 | 479 |  |  |
| 963 | 478 |  |  |
| 964 | 479 |  |  |
| 965 | 483 |  |  |
| 966 | 480 |  |  |
| 967 | 479 |  |  |
| 968 | 477 |  |  |
| 969 | 479 |  |  |
| 970 | 482 |  |  |
| 971 | 480 |  |  |
| 972 | 478 |  |  |
| 973 | 479 |  |  |
| 974 | 482 |  |  |
| 975 | 480 |  |  |
| 976 | 477 |  |  |
| 977 | 480 |  |  |
| 978 | 482 |  |  |
| 979 | 479 |  |  |
| 980 | 478 |  |  |
| 981 | 477 |  |  |
| 982 | 480 |  |  |
| 983 | 482 |  |  |
| 984 | 479 |  |  |
| 985 | 478 |  |  |
| 986 | 477 |  |  |
| 987 | 478 |  |  |
| 988 | 479 |  |  |
| 989 | 480 |  |  |
| 990 | 481 |  |  |
| 991 | 479 |  |  |
| 992 | 480 |  |  |
| 993 | 481 |  |  |
| 994 | 478 |  |  |
| 995 | 479 |  |  |
| 996 | 477 |  |  |
| 997 | 480 |  |  |
| 998 | 478 |  |  |
| 999 | 479 |  |  |
| 1000 | 477 |  |  |
| 1001 | 478 |  |  |
